# Sesamin protects SH-SY5Y cells against mechanical stretch injury and promoting cell survival

**DOI:** 10.1186/s12868-017-0378-8

**Published:** 2017-08-07

**Authors:** Zhiming Xu, Yingliang Liu, Dianxu Yang, Fang Yuan, Jun Ding, Hao Chen, Hengli Tian

**Affiliations:** Department of Neurosurgery, Shanghai Jiao Tong University Affiliated Sixth People’s Hospital, NO. 600, Yi Shan Road, Xuhui District, Shanghai, 200030 China

**Keywords:** Sesamin, Apoptosis, Oxidative stress, Mechanical stretch injury

## Abstract

**Background:**

Sesamin is a well-known antioxidant extracted from sesame seeds that exhibits various curative effects. The present study investigated whether sesamin would protect neuroblastoma SH-SY5Y cells against mechanical stretch injury-induced increases in reactive oxygen species (ROS) and apoptosis. Additionally, the mechanisms underlying these actives were investigated. Following exposure to mechanical stretch injury, cells were incubated for further investigations. Lactate dehydrogenase and Cell Counting Kit-8 assays were used to assess cell viability, and a terminal deoxynucleotidyl transferase dUTP nick end labeling assay and flow cytometric analysis were performed to evaluate changes in mitochondrial membrane potential (ΔΨm). Furthermore, intracellular levels of ROS production were measured by 20, 70-dichlorofluorescein diacetate staining, the mRNA levels of matrix metallopeptidase 9 (MMP-9) were evaluated using real-time polymerase chain reaction analysis, and the determinations had also been made on related proteins by Western blot analysis.

**Results:**

Exposure to mechanical stretch injury significantly decreased cell viability but this decrease was attenuated by pretreatment with sesamin (50 μM). Sesamin also significantly inhibited mechanical stretch injury-induced increases in intracellular ROS production, attenuated declines in ΔΨm, diminished the expressions of pro-apoptotic proteins, and decreased cell apoptosis. Stretch injury increased Bax and cleaved caspase 3 levels, enhanced the gene expression of MMP-9, increased the phosphorylation levels of Akt, p38, and JNK and decreased Bcl-2 levels in the cells. However, pretreatment with sesamin reduced the mechanical stretch injury-induced overexpression of MMP-9.

**Conclusions:**

Sesamin protected SH-SY5Y cells against stretch injury by attenuating increases in ROS levels and suppressing apoptosis. Accordingly, sesamin seems to be a potentially therapeutic agent in the treatment of traumatic brain injury.

**Electronic supplementary material:**

The online version of this article (doi:10.1186/s12868-017-0378-8) contains supplementary material, which is available to authorized users.

## Background

Traumatic brain injury (TBI) is a primary cause of deaths worldwide, surviving patients may also suffer from neuropsychiatric illnesses. Although substantial progresses have been achieved in the field of emergency surgery, treatment against TBI-induced neuron loss which would lead to neurological dysfunction closely related to hemiplegia, hypomnesia, Alzheimer’s disease (AD) have not been improved considerably yet. A Cell Injury Controller unit (Virginia Commonwealth University) would be utilized to perform rapid stretch, and the signaling response of cells was investigated consequently [1, 2]. It had been demonstrated that multi-impairments would occur after mechanical stretch-induced injury, delivery of a stretching pulse would cause increase in cytosolic calcium levels in neuronal and glial cells. In mixed neuronal-glial cultures, mechanical stretch injury evoked a “stretch-induced delayed depolarization”, which may relate to decrease in Na^+^, K^+^-ATPase activity [3, 4]. Mechanical stretch injury may produce many of the post-traumatic responses which had been observed in vivo, including intracellular impairments on mitochondrial and cytoskeletal elements [5, 6].

Sesamin is the major lignan obtained from sesame oil and may protect against various injuries induced by hypoxia and H_2_O_2_ [7, 8]. Sesamin may also alleviate oxidative stress and mortality under conditions of kainic acid-induced status epilepticus by inhibiting the activation of mitogen-activated protein kinases (MAPK) and cyclooxygenase (COX)-2 [9]. Furthermore, treatment with sesamin also inhibits the activation of BV2 cells and attenuates increases in cytokine production following exposure to lipopolysaccharide (LPS) [10, 11].

Certain levels of reactive oxygen species (ROS) are necessary for normal cellular function, but overproduction of these molecules alters the natural balance and can threaten cell viability. Although ROS, which include peroxides, superoxides, hydroxyl radicals, and singlet oxygen molecules, are primarily produced by microglia in the central nervous system (CNS), these molecules can also be produced by neurons and astrocytes and contribute to imbalances in ROS levels to threaten cell survival [12]. It had also been reported that the PI3 K/Akt survival signaling and the JNK/p38 apoptotic signaling pathway were activated in neurons following oxidative stress [17, 18].

Because sesamin plays a protective role both in vivo and in vitro, its anti-ROS activity is likely a key factor contributing to neuroprotection after TBI. Thus, the present study investigated the effects of sesamin in an in vitro model of TBI and attempted to determine the underlying mechanisms supporting this process.

## Methods

### Cell lines and reagents

**Fig. 1:**
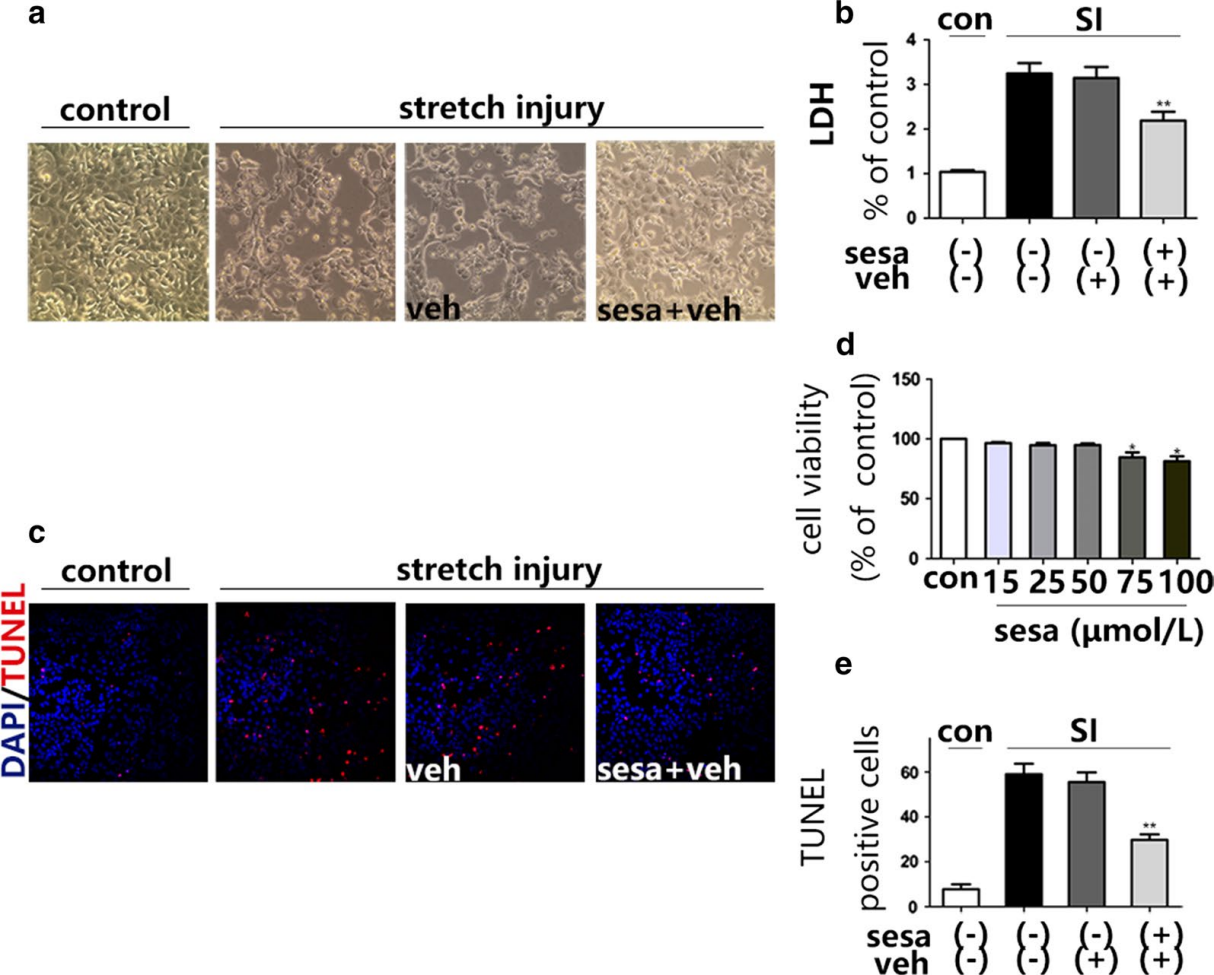
Protective effects of sesamin (sesa) against mechanical stretch injury-induced cytotoxicity. **a** At 24 h after mecnanical stretch injury, SH-SY5Y cells were analyzed for morphological changes at ×200; **b** *Bar graph* showing LDH levels in the extracellular fluid after stretch injury; **c** DNA damage detected by TUNEL assay; **d** SH-SY5Y cells were treated with different concentrations of seamin. Cell viability was estimated using CCK-8 assays. **e** *Bar graph* showing the percentage of TUNEL-positive cells. Mean ± SEM, *n* = 6, *p < 0.05, **p < 0.01, compared with vehicle (veh) treated cells

**Fig. 2:**
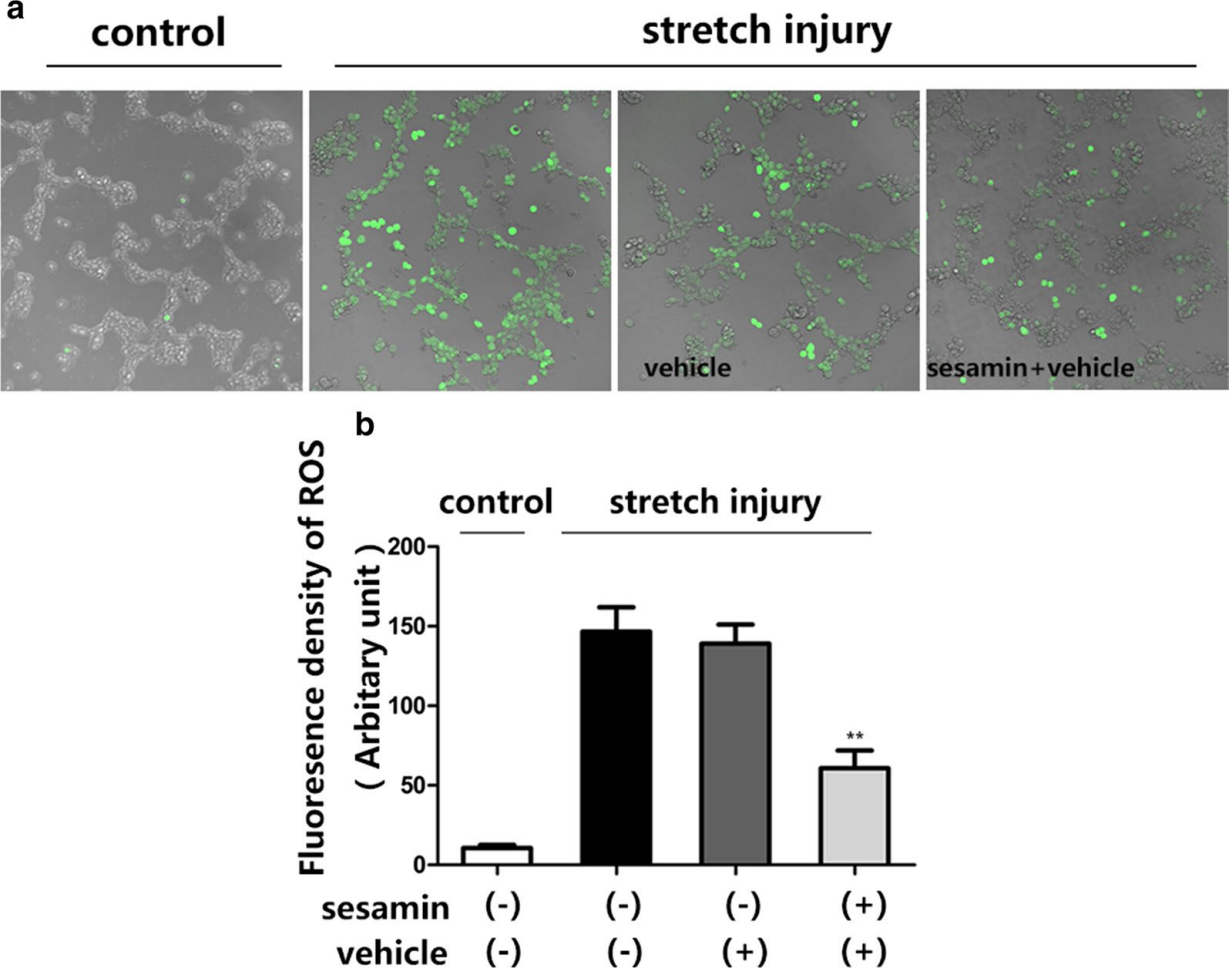
Sesamin decreased intracellular ROS generation after stretch injury. **a** Intracellular ROS levels were detected using DCF-DA. **b** *Bar graph* showing the quantitative results as a percentage of the control. Mean ± SEM, *n* = 6, **p < 0.01, compared with vehicle treated cells

SH-SY5Y cells were acquired from the Cell Bank of the Shanghai Institute of Cell Biology and Biochemistry, Chinese Academy of Sciences (Shanghai, China). Human neuroblastoma SH-SY5Y cell lines were cultured in Dulbecco’s Modified Eagle’s Medium (DMEM; Gibco Laboratories; Grand Island, NY, USA) supplemented with 10% fetal bovine serum (FBS), 100 μg/mL streptomycin, and 100 μg/mL penicillin. The cultures were then transferred to an anaerobic chamber infused with a gas mixture containing 5% CO_2_ and 95% N_2_. For the experiments, the cells were seeded at a density of 0.14–0.6 × 10^5^/cm^2^ on collagen-coated glass cover slips or BioFlex elastic membrane supports (Flexcell International Corp.; Burlington, NC). To minimize the influence of unconsolidated attachment to the substratum, the cells were seeded on poly-d-lysine (PDL, Sigma-Aldrich, St. Louis, MO, USA) coated Flex Plates. Pure sesamin (Aladdin Corp; Shanghai, China) was dissolved in DMSO (Sigma Aldrich) as stock buffer and the final concentration of DMSO in culture media was diluted to 1‰. We had determined primary neurons (Cells were used for experiments within 17 days after removal from cerebral cortices of C57BL/6 mice) and SH-SY5Y cells cellular ATP levels following mechanical stretch injury. Based on data derived from the test, SH-SY5Y cells had been chosen in this study (data had been shown in Additional files 1, 2: Figures 1 and 2).

### Cell Counting Kit-8 (CCK-8) assay

5 × 10^3^ SH-SY5Y cells were seeded per well in 96-well plates. 14–16 h later, cells were incubated with differ-ent concentrations of sesamin for another 24 h. Cell viability was determined using 2-(2-methoxy-4-nitrophenyl)-3-(4-nitrophenyl)-5-(2,4-disulfophenyl)-2*H*-tet-razolium (WST-8) and monosodium salt with a CCK-8 assay (Beyotime Biotechnology, China) accord-ing to the manufacturer’s instructions. The absorbance was determined at 450 nm using a spectrophotometer.

### Mechanical stretch injury

In preparation for mechanical injury, the SH-SY5Y cells were seeded on collagen- coated silastic membranes of BioFlex six-well plates. Next, a biaxial stretch was produced by 50-ms burst of nitrogen gas using the Cell Injury Controller II system (Virginia Commonwealth University) which induced a downward deformation of the silastic membrane and adherent cells. The SH-SY5Y cells were underwent stretch injury via a 6.5-mm deformation of silastic membranes and then cultured for 24 h. The degree of deformation is supposed to be corresponding to the mechanical stress range imposed on human brain injury following rotational acceleration-deceleration injuries [2, 13].

### Lactate dehydrogenase (LDH) assay

A lactate dehydrogenase (LDH) detection kit (Roche Applied Science; Basel, Switzerland) was applied to evaluate the levels of cytotoxicity. The SH-SY5Y cells were cultured in 6-well plates. Cells were pretreated with sesamin (50 μM) for 1 h before mechanical cell injury. LDH activity was measured in the culture media after 24 h. The absorbance at 490 nm of samples were evaluated using a spectrophotometer (BioTek, Winooski, VT, USA). Results were expressed as percentage of the control.

### Determination of DNA fragmentation

To assess DNA fragmentation, a TUNEL staining assay was performed [14, 15]. SH-SY5Y cells were treated with sesamin an hour before stretch injury, after 24 h incubation, cells were fixed with 4% paraformaldehyde, a Cell Death Detection kit (Roche) was utilized to detect DNA fragmentation. The levels of DNA fragmentation was identified by fluorescence microscopy.

### Measurement of ROS levels in SH-SY5Y cells after mechanical injury

20, 70-dichlorofluorescein diacetate (DCF-DA) staining was used to determine intracellular ROS levels in SH-SY5Y cells. The cells were pre-incubated with sesamin(50 μM) for 1 h followed by mechanical stretch injury. Next, the cells were washed with Hank’s balanced salt solution (HBSS without phenol red), loaded with 20 mM DCF-DA for 30 min at 37 °C and washed again. To quantify the fluorescence of DCF-DA, a fluorescence microscopy was utilized and setted at excitation wavelengths of 488 nm and emission wavelengths of 525 nm. ROS production was displayed as percentage of the control.

### Fluorescence-activated cell sorting (FACS) based determinations of mitochondrial membrane potential (ΔΨm)

The mitochondrial membrane potential (ΔΨm) of SH-SY5Y cell was determined by JC-1 (5, 5′, 6, 6′-tetrachloro-1, 1′, 3, 3′-tetraethylbenzimidazolylcarbocyanine iodide; Enzo Life Sciences; PA, USA). JC-1 accumulates in mitochondria matrix in healthy cells which have higher ΔΨm and emit red fluorescence at the wavelength of 590 nm. SH-SY5Y cells were digested with 0.25% trypsin, cells were washed and resuspended in PBS. 10 μg/mL JC-1 was added, and incubated in thermotank 37 °C away from light, the cells were analyzed by FACS Aria II (BD Biosciences) without delay. Both red and green fluorescence were analyzed by FACS, the ratio between the red fluorescence and the green fluorescence was introduced to measureΔΨm of SH-SY5Y cells.

### Real-time polymerase chain reaction analysis

Total RNA samples were extracted from SH-SY5Y cells through the TRIzol reagent (Invitrogen; Carlsbad, CA, USA) and then reverse-transcribed to cDNA by a PrimeScript RT reagent kit (TaKaRa). The oligonucleotide primers used to amplify the target genes were as follows: MMP-9 antisense, 5′-GTCGCCCTCAAAGGTTTGGAAT-3′; MMP-9 sense, 5′-GTGCTGGGCTGCTGCTTTGCT-3′; and glyceraldehyde 3-phosphate dehydrogenase (GAPDH) antisense, 5′-AGTGATGGCATGGACTGTGGTCAT-3′; GAPDH sense, 5′-ACCCCTTCATTGACCTCAACTACA-3′. A quantitative rt-PCR procedure was carried out with an ABI 7900HT PCR system using SYBR Premix Ex Taq (TaKaRa).

### Western blot analysis

For Western blot analysis, SH-SY5Y cells were lysed in radioimmunoprecipitation assay buffer (Millipore; Bedford, MA, USA), 1 mmol/L of PMSF protease inhibitor (Thermo; Waltham, MA, USA), Halt™ protease inhibitor cocktail and phosphatase inhibitor (Thermo) were added before start. Same amount of protein samples which had been denatured were loaded onto the 10% resolving gel (Promoton; Shanghai, China) for electrophoresis, the proteins were transferred onto a nitrocellulose membrane (Whatman; Piscataway, NJ, USA) subsequently. The membranes loaded with proteins were incubated with primary antibodies overnight at 4 °C after blocking with 5% non-fat milk for an hour. The dilutions of primary antibodies were as following: p-Akt and Akt (1:500, Cat. NO. 4060 and 4691), p-JNK and JNK (1:500, Cat. NO. 4668 and 9258), p-p38 and p38 (1:500, Cat. NO. 4511 and 8690), cleaved caspase 3 and caspase 3 (1:500, Cat. NO. 9664 and 9665), Bcl-2, Bax and GAPDH (1:1000, Cat. NO. 15071, 2774 and 5174, Cell Signaling Technology; Beverly, MA, USA), β-actin (1:1000, Cat. NO. sc-58673, Santa Cruz Technology; Santa Cruz, CA, USA) and MMP-9 (1:1000, Cat. NO. 10375-2-AP, Proteintech Group, Inc. Rosemont, IL, USA). After washing, appropriate horseradish peroxidase (HRP)-conjugated secondary antibodies were selected to incubate nitrocellulose membrane for an hour at room temperature, the membrane then reacted with an enhanced chemiluminescence substrate (Pierce; Rockford, IL, USA) away from light.

### Statistical analysis

Data were presented as mean ± standard error of the mean (SEM). Levene’s test and t-tests were applied to compare the equality of variance. Histograms presented in article were created by GraphPad Prism 6 (GraphPad Software; San Diego, CA). SPSS 20.0 for Windows (SPSS Inc., Chicago, IL) was used to analyse data and there was no statistical significance unless *p* values <0.05.

## Results

### Cytotoxicity of different concentrations of sesamin on SH-SY5Y cells

**C**CK-8 assays were applied to inspect cytotoxicity of 5, 25, 50, 75 and 100 μM of sesamin on SH-SY5Y cells after 24 h incubation. The viability of cells incubated with sesamin at 15, 25, 50, 75 and 100 μM for 24 h were 96.5 ± 1.7, 94.7.0 ± 3.7, 94.8 ± 2.8, 84.5 ± 8.3, and 81.3 ± 8.2% of the control values (Fig. 1d). The results showed that sesamin at 75 and 100 μM decreased cell viability com-pared to the untreated control group. There was no statistical significance in cell viability between untreated control group and 50 μM sesamin treated group, implying that the concentration of sesamin chosen in present study was not cytotoxic.

### Pretreatment of sesamin promoted the survival of SH-SY5Y cells after mechanical stretch injury

The effects of sesamin pretreatment on the survival of SH-SY5Y cells were determined with an extracellular LDH assay after the cells were incubated for 24 h with 50 μM of sesamin. Stretch injury increased the extracellular levels of LDH but sesamin enhanced the survival rate of SH-SY5Y cells, relative LDH levels of sesa-/veh- group and sesamin group were 3.24 ± 0.235, 2.188 ± 0.2, *p* = 0.0091, F = 1.382 (Fig. 1a, b).

### Sesamin suppressed oxidative stress and oxidative stress-mediated cellular signaling in SH-SY5Y cells exposed to mechanical stretch injury

**Fig. 3:**
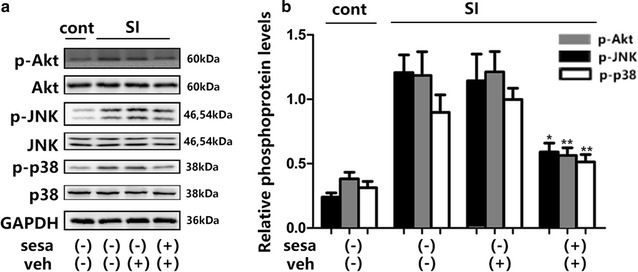
Pretreatment of sesamin downregulated protein levels in ROS-mediated cell signaling pathways after stretch injury (SI). **a** Phosphorylation levels of Akt, JNK, and p38 were detected and analyzed using Western blot and densitometric analyses. **b** *Bar graph* showing the quantitative results as a percentage of the control. Mean ± SEM, *n* = 5, *p < 0.05, **p < 0.01, compared with vehicle treated cells

It had been reported that mchanical stretch injury not only impaired mitochondrial function, but also increased ROS productions in cells [16]. In this study, the cells were pretreated with sesamin for an hour and the levels of ROS were quantified using DCF-DA staining. Stretch injury significantly increased the intracellular levels of ROS in SH-SY5Y cells but pretreatment with sesamin (50 μM) decreased ROS production (Fig. 2). Sesamin considerably decreased phosphorylation levels of Akt and JNK/p38 in SH-SY5Y cells that were exposed to stretch injury, relative protein levels of Akt, JNK and p38 in vehicle group were 1.143 ± 0.208, 1.213 ± 0.159, 0.998 ± 0.0886, relative phosphorylated protein levels were 0.59 ± 0.069 (*p* = 0.045, F = 8.963), 0.0562 ± 0.06 (*p* = 0.0086, F = 6.934), 0.5125 ± 0.058 (*p* = 0.0038, F = 2.295) (Fig. 3).

**Fig. 4:**
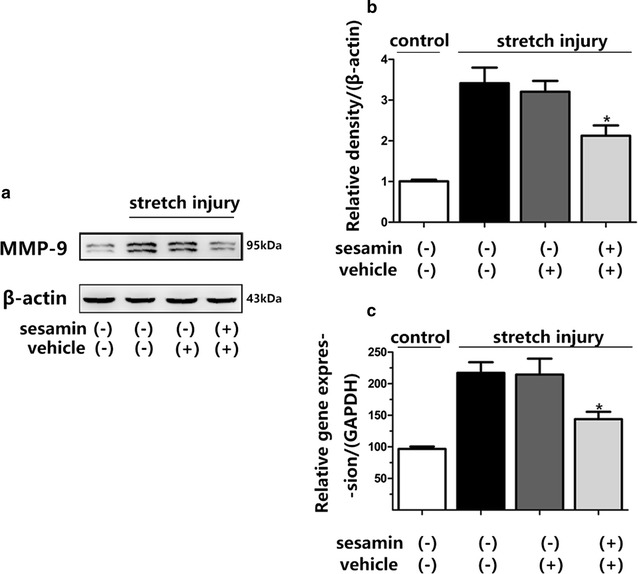
Sesamin inhibited overexpression of MMP-9 mRNA and protein levels. **a** Representative results of MMP-9 expression after stretch injury. **b**, **c** *Bar graphs* showing the quantification of MMP-9 expression. Mean ± SEM, *n* = 5, *p < 0.05, compared with vehicle treated cells

A lot of researchs conducted on neuronal cells indicated that MMP-9 was an essential role in a variety of disease models [19–21]. Therefore, the effects of stretch injury and sesamin pretreatment on the expression of MMP-9 were assessed using Western blot and RT-PCR analyses in the present study.Pretreatment with sesamin significantly decreased the overexpression of MMP-9 after stretch injury in SH-SY5Y cells, relative protein levels of MMP-9 were 3.205 ± 0.2658 versus 2.125 ± 0.2546, *p* = 0.0262, F = 1.09 (vehicle vs sesamin), and relative gene expression of MMP-9 decreased 30.3% (Fig. 4). Together, the present findings indicated that sesamin treatment protected against mechanical injury-induced SH-SY5Y cell death, this protective effect may relate to inhibition phosphorylation levels in Akt and JNK/p38 signaling pathways and suppression the expression of MMP-9.

### Sesamin treatment reduced stretch injury-induced apoptosis in SH-SY5Y cells

Stretch injury leads to mitochondrial impairments in astrocytes and neurons [2]. Accordingly, this study estimated the effects of oxidative stress in relation to mitochondrial dysfunction and apoptosis following mechanical stretch injury to SH-SY5Y cells. A TUNEL assay was conducted to determine the effects of sesamin on oxidative stress-induced DNA fragmentation. SH-SY5Y cells which had been exposed to stretch injury were vulnerable to mitochondrion-induced impairments in DNA, pretreatment with sesamin significantly protected the cells against this DNA damage, however (Fig. 1c, e).

**Fig. 5:**
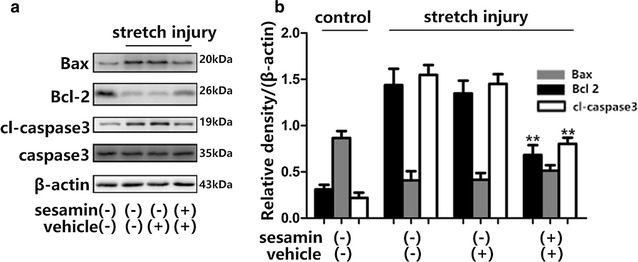
Sesamin protected SH-SY5Y cells via alterations in anti-apoptotic and pro-apoptotic proteins after stretch injury. **a** Activation of apoptotic molecules were detected using Bax, Bcl-2 and cleaved caspase 3. **b** Quantified with a Western blot analysis. Mean ± SEM, *n* = 5, *p < 0.05, **p < 0.01, compared with vehicle treated cells

Next, the effects of mechanical stretch injury on oxidative stress-induced apoptosis signaling pathways were evaluated. Mechanical stretch injury decreased levels of the anti-apoptotic protein and increased the levels of pro-apoptotic proteins. Sesamin decreased the levels of Bax and cleaved caspase 3 (vehicle vs sesamin, Bax: 1.348 ± 0.1391 vs 0.6825 ± 0.1073, *p* = 0.0091, F = 1.678; cleaved caspase 3: 1.45 ± 0.1062 vs 0. 8025 ± 0.06613, *p* = 0.0021, F = 2.58), but pretreatment with sesamin decreased the protein levels of cleaved caspase 3 and Bax while the expression of Bcl-2was aggravated (Fig. 5). Critically, mitochondrial permeability was partly stabilized by Bcl-2 via its interaction with Bax.

**Fig. 6:**
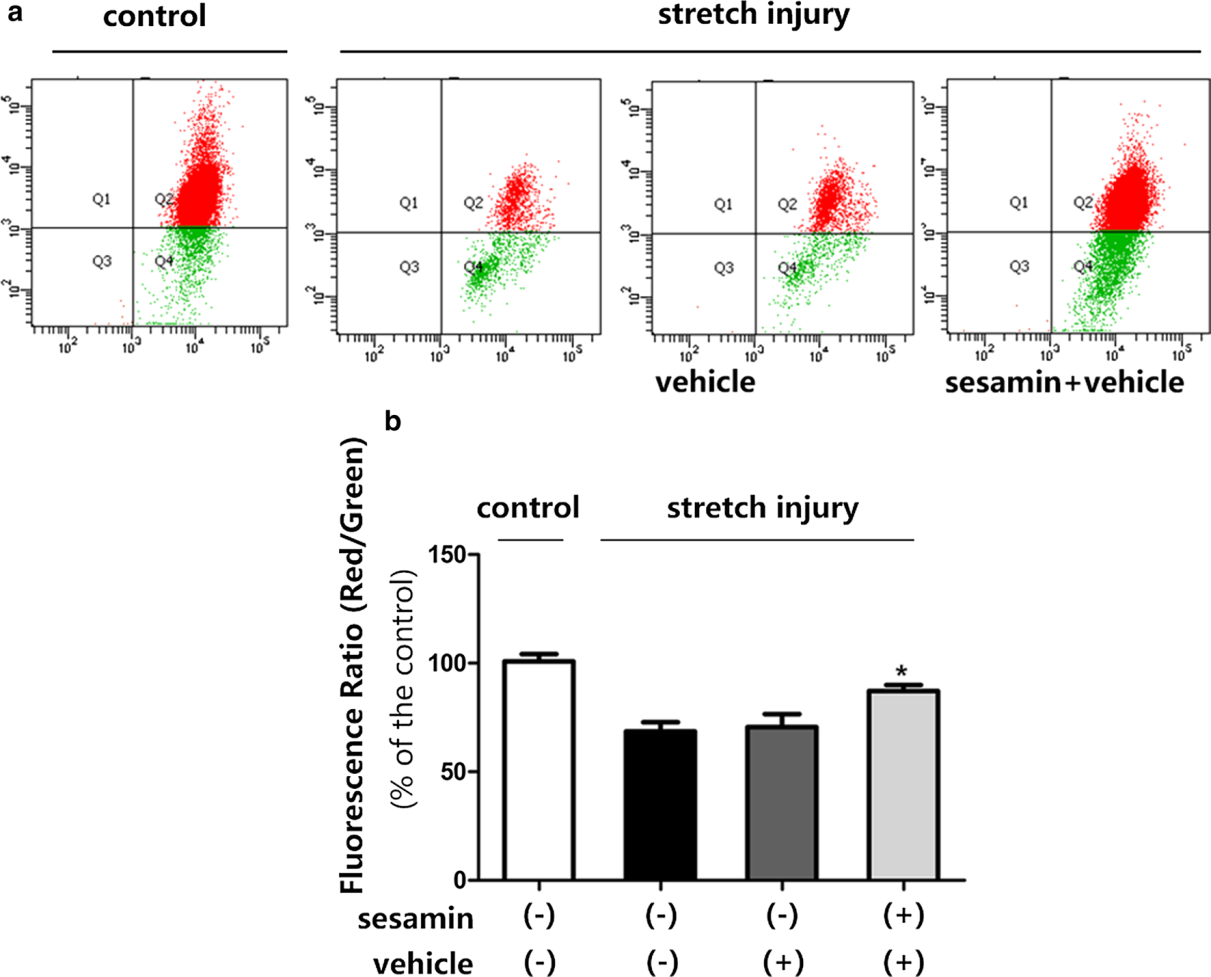
Sesamin significantly increased the ΔΨm in SH-SY5Y cells exposed to stretch injury. Cells were pretreated with either sesamin or vehicle then exposed to stretch injury. **a** After 6 h, cells were collected for flow cytometry test. **b** *Bar graphs* showing the JC-1 aggregate/JC-1 monomer ratios (*red* fluorescence/*green* fluorescence ratios) normalized to baseline. Mean ± SEM, *n* = 5, *p = 0.0102, compared with vehicle treated cells

Consequently, the potentially protective role of sesamin in the oxidative stress mediated disruption of mitochondrial membrane following mechanical stretch injury was assessed. The ΔΨm is generated by the special configuration of the outer and inner mitochondrial membranes; this potential decreases and membrane instability increases under conditions of mitochondrial dysfunction. The present findings demonstrated that the application of mechanical stretch injury to SH-SY5Y cells influenced the expressions of anti-apoptotic and pro-apoptotic proteins that are closely related to mitochondrial function. Therefore, a FACS-based JC-1 assay was conducted to determine ΔΨm the of the SH-SY5Y cells. Mechanical stretch injury resulted in mitochondrial depolarization, which in turn may influence apoptotic process in SH-SY5 cells. Mechanical stretch injury decreased ΔΨm of the SH-SY5Y cells, pretreatment with sesamin resulted in an approximately 40% increase in ΔΨm (Fig. 6).

## Discussion

Our study proved that sesamin was able to alleviate mechanical stretch injury-induced cytotoxicity in SH-SY5Y cells. The potentially protective effects of sesamin against mechanical stretch injury-induced impairments may be relevant to attenuation of apoptosis and ROS levels.

Mechanical stretch injury, which was developed and characterized by Ellis and coworkers, had been used to study the effects of trauma on neurons and astrocytes in vitro [2, 22]. It has also been used to explore cellular alterations including increased plasma membrane permeability, phospholipase C activation, arachidonic acid release and membrane depolarization [1, 3, 4, 23]. Here, we explored the cytotoxicity of mechancial stretch injury on SH-SY5Y cells. The results demonstrated that stretch injury decreased cell viability, yet sesamin attenuated this reduc-tion. Previous studies had revealed that activation of apoptotic pathways involved in stretch injury-induced neurotoxicity [24]. The Bcl-2 and caspase family proteins took part in regulation of intrinsic apoptotic pathway. It has been proposed that mechanical stretch injury would induce increase in ROS forma-tion and cellular apoptosis [25–27]. The neuroprotective effects against mechanical stretch injury may be relevant to suppression of pro-apoptotic Bax expression or caspase-3 activity and the upregulation of anti-apoptotic Bcl-2 expression. In accordance with previous results, in this study, the Bax/Bcl-2 ratio and caspase-3 activity elevated in SH-SY5Y cells following mechanical stretch injury. Sesamin may reduce these undesirable effects which indicated the protective role of sesamin in SH-SY5Y cells via alleviating apoptosis when cells were exposed to mechanical stretch injury.

Following TBI, neuronal loss is characterized by oxidative stress reaction, mitochondrial dysfunction, neurotoxicity, and neuroinflammation [25, 28]. Pretreatment with sesamin inhibited oxidative stress-mediated cellular processes in SH-SY5Y cells exposed to mechanical stretch injury. The neuronal SH-SY5Y cell line is a well known, reliable, and efficient paradigm for the investigation of ROS and neuroprotection [25, 29]. Additionally, because the SH-SY5Ycell model has never been used to evaluate the effects of sesamin on damage induced by stretch injury previously, the dosage used in the present study was selected base on previous studies and our own research [10, 30]. Our findings demonstrated that mechanical stretch injury to SH-SY5Y cells resulted in the production of intracellular ROS and cellular death and sesamin attenuated disadvantages after stretch injury.

Depending on their levels, ROS play a variety of roles in several cellular processes. Moderate levels of ROS have a positive influence on cell function, whereas high levels of ROS accelerate the progression of neurodegenerative diseases, inflammatory disorders, and cancers that can lead to cell death [16, 31]. Malignant ROS levels have been observed in neurons and endothelial cells associated with traumatic injuries [32–34]. The present results support the theory that sesamin decreases intracellular ROS levels and suppresses the expression of ROS-related proteins.

Healthy cells generate relatively high ΔΨm, JC-1 accumulates in mitochondrial matrix and forms J-aggregates which emit red fluorescence. When cells have a decreased ΔΨm, additional JC-1 monomers are generated and emit green fluorescence. Accordingly, decrease in the ΔΨm after mitochondrial dysfunction leads to the accumulation of ROS in cells [35]. Recently, an increasing number of studies have identified positive correlations between intake of certain foods that contain high levels of natural antioxidants and various diseases, including stroke and coronary heart disorders [36]. The protective effects of sesamin in cells which were exposed to mechanical stretch injury were primarily due to its antioxidant effects, reversal of changes in the ΔΨm, inhibition of the phosphorylation of Akt and JNK/p38, and the decreased apoptotic expression of signaling pathways.

## Conclusion

The present findings suggest that the protective role of sesamin in SH-SY5Y cells that were exposed to mechanical stretch injury was mainly due to its anti-ROS and anti-apoptotic activities. Therefore, sesamin appears to have potentially therapeutic values which would alleviate mitochondrial dysfunction, oxidative stress, and cytotoxicity associated with mechanical stretch injury in vitro model of TBI.

## Additional files

**Additional file 1: Figure 1.** ATP content was detected by ATP assy kit (Beyotime, China) at 15 min after injury in neuronal cultures, cell injury controller transiently deformed the silastic membrane of the Flex Plate and adherent cells to varying degrees (5.7 and 6.5 mm) controlled by the pulse pressure. Neurons were injured and lysed at 15 min postinjury, and ATP was quantified using a luminometer. Controls consisted of uninjured cells. Data for neurons were from four different experiments. *p* > 0.05 versus control, mechanical stretch injury did not lead to a significant decline in cellular ATP levels.

**Additional file 2: Figure 2.** ATP content was detected by ATP assy kit (Beyotime, China) at 15 min after injury in SH-SY5Y cells, cells were exposed to varying degree damage as neurons. SH-SY5Y cells were injured and lysed at 15 min postinjury, and ATP was quantified using a luminometer. Controls consisted of uninjured cells. 5.5 mm deformation did not lead to a significant decline in cellular ATP levels, *p* > 0.05 versus control, however, 6.5 mm deformation significantly decreased cellular ATP levels, *p < 0.05 versus control.

## Abbreviations

ROS: reactive oxygen species
LDH: lactate dehydrogenase
CCK-8: Cell Counting Kit-8
MMP-9: matrix metallopeptidase 9
RT-PCR: real-time polymerase chain reaction
DMSO: dimethyl sulfoxide
ΔΨm: mitochondrial membrane potential
JC-1: 5, 5′, 6, 6′-tetrachloro-1, 1′, 3, 3′-tetraethylbenzimidazolylcarbocyanine iodide
FACS: fluorescence-activated cell sorting
TUNEL: terminal deoxynucleotidyl transferase dUTP nick end labeling
DCF-DA: 20,70-dichlorofluorescein diacetate
