# Supplementary material for: Sesamin protects SH-SY5Y cells against mechanical stretch injury and promoting cell survival
Source: BMC Neurosci. 2017 Aug 7;18:57. doi: 10.1186/s12868-017-0378-8 (PMC5547510; doi:10.1186/s12868-017-0378-8)
Supplement: Supplementary file 1 — Additional file 1: Figure 1. ATP content was detected by ATP assy kit (Beyotime, China) at 15 min after injury in neuronal cultures, cell injury controller transiently deformed the silastic membrane of the Flex Plate and adherent cells to varying degrees (5.7 and 6.5 mm) controlled by the pulse pressure. Neurons were injured and lysed at 15 min postinjury, and ATP was quantified using a luminometer. Controls consisted of uninjured cells. Data for neurons were from four different experiments. p > 0.05 versus control, mechanical stretch injury did not lead to a significant decline in cellular ATP levels. [file 12868_2017_378_MOESM1_ESM.docx]

Supplementary Fig. 1 ATP content was detected by ATP assy kit (Beyotime, China) at 15 min after injury in neuronal cultures, cell injury controller transiently deformed the silastic membrane of the Flex Plate and adherent cells to varying degrees (5.7 mm and 6.5 mm) controlled by the pulse pressure. Neurons were injured and lysed at 15 min postinjury, and ATP was quantified using a luminometer. Controls consisted of uninjured cells. Data for neurons were from four different experiments. *p* > 0.05 versus control, mechanical stretch injury did not lead to a significant decline in cellular ATP levels.
