# Supplementary material for: Sesamin protects SH-SY5Y cells against mechanical stretch injury and promoting cell survival
Source: BMC Neurosci. 2017 Aug 7;18:57. doi: 10.1186/s12868-017-0378-8 (PMC5547510; doi:10.1186/s12868-017-0378-8)
Supplement: Supplementary file 2 — Additional file 2: Figure 2. ATP content was detected by ATP assy kit (Beyotime, China) at 15 min after injury in SH-SY5Y cells, cells were exposed to varying degree damage as neurons. SH-SY5Y cells were injured and lysed at 15 min postinjury, and ATP was quantified using a luminometer. Controls consisted of uninjured cells. 5.5 mm deformation did not lead to a significant decline in cellular ATP levels, p > 0.05 versus control, however, 6.5 mm deformation significantly decreased cellular ATP levels, *p < 0.05 versus control. [file 12868_2017_378_MOESM2_ESM.docx]

Supplementary Fig. 2 ATP content was detected by ATP assy kit (Beyotime, China) at 15 min after injury in SH-SY5Y cells, cells were exposed to varying degree damage as neurons. SH-SY5Y cells were injured and lysed at 15 min postinjury, and ATP was quantified using a luminometer. Controls consisted of uninjured cells. 5.5 mm deformation did not lead to a significant decline in cellular ATP levels, *p* > 0.05 versus control, however, 6.5 mm deformation significantly decreased cellular ATP levels, **p* < 0.05 versus control.
